# Transient introduction of human telomerase mRNA improves hallmarks of progeria cells

**DOI:** 10.1111/acel.12979

**Published:** 2019-05-31

**Authors:** Yanhui Li, Gang Zhou, Ivone G. Bruno, Ning Zhang, Sei Sho, Enzo Tedone, Tsung‐Po Lai, John P. Cooke, Jerry W. Shay

**Affiliations:** ^1^ Department of Cell Biology UT Southwestern Medical Center Dallas Texas; ^2^ Department of Cardiovascular Sciences Houston Methodist Research Institute Houston Texas; ^3^ Lonza Walkersville Inc. Walkersville Maryland

**Keywords:** aging, Hutchinson‐Gilford progeria syndrome, RNA therapy, telomerase, telomeres

## Abstract

Hutchinson–Gilford progeria syndrome (HGPS) is characterized by accelerated senescence due to a de novo mutation in the *LMNA* gene. The mutation produces an abnormal lamin A protein called progerin that lacks the splice site necessary to remove a farnesylated domain. Subsequently, progerin accumulates in the nuclear envelope, disrupting nuclear architecture, chromatin organization, and gene expression. These alterations are often associated with rapid telomere erosion and cellular aging. Here, we further characterize the cellular and molecular abnormalities in HGPS cells and report a significant reversal of some of these abnormalities by introduction of in vitro transcribed and purified human telomerase (hTERT) mRNA. There is intra‐individual heterogeneity of expression of telomere‐associated proteins DNA PKcs/Ku70/Ku80, with low‐expressing cells having shorter telomeres. In addition, the loss of the heterochromatin marker H3K9me3 in progeria is associated with accelerated telomere erosion. In HGPS cell lines characterized by short telomeres, transient transfections with hTERT mRNA increase telomere length, increase expression of telomere‐associated proteins, increase proliferative capacity and cellular lifespan, and reverse manifestations of cellular senescence as assessed by β‐galactosidase expression and secretion of inflammatory cytokines. Unexpectedly, mRNA hTERT also improves nuclear morphology. In combination with the farnesyltransferase inhibitor (FTI) lonafarnib, hTERT mRNA promotes HGPS cell proliferation. Our findings demonstrate transient expression of human telomerase in combination with FTIs could represent an improved therapeutic approach for HGPS.

## 1 INTRODUCTION

Hutchinson–Gilford progeria syndrome (HGPS) is a rare disorder of accelerated aging. In childhood, patients exhibit growth retardation, alopecia, reduced subcutaneous adipose tissue, osteoporosis, and sclerodermatous skin. These individuals often succumb to myocardial infarction or stroke, with an average lifespan of 15 years. The disease is due to an autosomal 1824 C to T mutation in the LMNA gene encoding lamin A, a nuclear membrane protein. The mutation results in a truncated and constitutively farnesylated lamin A called progerin (Eriksson et al., 2003). Progerin accumulates in the nuclear envelope and severely disrupts nuclear architecture and cellular function (Goldman et al., 2004). Farnesyltransferase inhibitors (FTIs) inhibit progerin farnesylation and the intercalation of progerin into the nuclear envelope (Capell et al., 2005). A clinical trial with the FTI lonafarnib implied that this therapy might ameliorate cardiovascular and bone disease in HGPS patients, but the benefit observed was modest (Gordon et al., 2012). Accordingly, there is an urgent need to develop new therapies for these children.

Human telomeres contain TTAGGG repeats and are capped with a six‐member protein shelterin complex and other telomere‐associated proteins such as DNA PKcs, Ku70, and Ku80 (Celli, Denchi, & Lange, 2006; de Lange, 2005). Telomeres undergo progressive shortening with cell divisions due in part to the “end‐replication problem,” a consequence of the inability of DNA polymerases to replicate fully the DNA lagging strand. In stem cells, the end‐replication problem is partially addressed by transient telomerase activation, an RNA‐dependent DNA polymerase, which adds telomeric repeats to telomere ends by copying a template sequence of the RNA component (*TERC*) (Shay, 2016). In addition, one of the shelterin proteins, TRF2, can interact with lamin a/c to facilitate the functional organization of chromosome ends (Wood et al., 2014) and this may tie telomere biology to the functional defect in HGPS.

Hutchinson–Gilford progeria syndrome is associated with accelerated telomere erosion (Decker, Chavez, Vulto, & Lansdorp, 2009). Telomere erosion will ultimately elicit a DNA damage response, resulting in irreversible growth arrest and senescence. The fundamental mechanism of rapid telomere erosion in HGPS patients remains elusive. This process is likely secondary to the accumulation of progerin in the nuclear inner membrane, where nuclear lamina physically and functionally interacts with telomeres (Chojnowski et al., 2015; Taddei, Hediger, Neumann, & Gasser, 2004). One possibility is that telomere erosion contributes to the accelerated senescence in these patients. Accordingly, we tested the hypothesis that transient expression of purified human telomerase (hTERT) mRNA might partially restore telomere length and reverse some of the senescent phenotypes of patient cells.

## 2 RESULTS

### 2.1 Telomere length variations in HGPS

**Figure 1:**
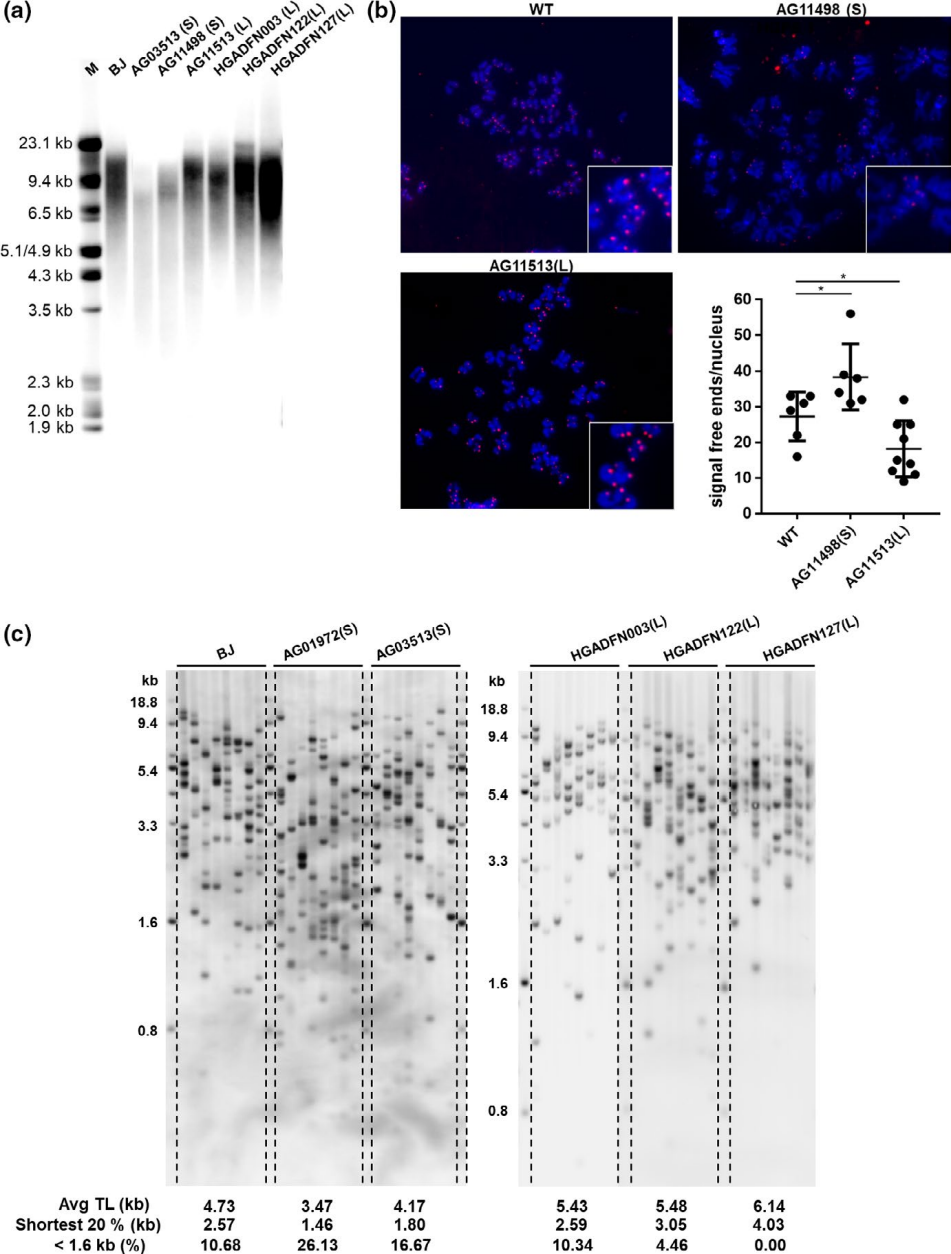
Heterogeneity of telomere length in Hutchinson–Gilford progeria syndrome patients. (a) Telomere length in BJ and progeria fibroblasts by terminal restriction fragment (TRF) assay. Mean telomere lengths: BJ (PD28), 12.7 kb; AG3513, 8.7 kb; AG11498, 10.5 kb; AG11513, 12.8 kb; HGADFN003, 12.3 kb; HGADFN122, 13 kb; HGADFN127, 12.1 kb. (b) Telomere‐FISH of BJ (PD28), AG11498, and AG11513 cells. 6–9 metaphases were analyzed per condition. *p < 0.05 (Student's *t* test). (c) Short telomeres distribution in BJ and progeria cells, as detected by TeSLA. L, long telomeres; S, short telomeres

The telomeres of HGPS patients are generally shorter for their chronological age (Decker et al., 2009). However, there is heterogeneity in average telomere length among HGPS patients, with ~29% of patients having normal or even longer telomeres (Li, Zhou, Bruno, & Cooke, 2017) (Figure S1). We explored the telomere length variations in HGPS fibroblast cells. Terminal restriction fragment (TRF) analysis revealed reduced intensity and size distribution of the telomere smears in some, but not all HGPS cell lines (Figure 1a; Figure S2). Telomere‐FISH further validated diminished telomere signals in patient AG11498 cells (short telomeres), but not in AG11513 cells (long telomeres), compared with cells from a normal subject. We did not observe telomere fusions in these samples (Figure 1b).

It is widely accepted that the shortest telomeres trigger cellular replicative senescence. Critically short telomeres activate DNA damage responses leading to cell cycle arrest (Zou, Sfeir, Gryaznov, Shay, & Wright, 2004). We analyzed the telomere distribution spectrum of HGPS cell lines using the Telomere Shortest Length Assay (TeSLA). TeSLA measures both the average telomere length and quantitatively provide information on the shortest telomeres (<1.6 kb) that other telomere measurement methods cannot visualize (Lai et al., 2017). We found short telomere HGPS cells had reduced mean telomere lengths (4.7 kb in BJ; 3.47 kb in AG01972; 4.17 kb in AG03513) and more of the shortest telomeres below 1.6 kb compared with control cells (10.68% in BJ; 26.13% in AG01972; 16.67% in AG03513). In contrast, long telomere HGPS cells had a normal short telomere distribution below 1.6 kb (10.34% in HGADFN003; 4.46% in HGADFN122; 0% in HGADFN127; Figure 1c). While the different patient cells were in culture for a similar number of population doublings, as expected the short telomere patients were older (13 and 14 years) compared with the younger long telomere patients (2, 4, and 5 years), indicating the shortest telomeres accumulate at an earlier age in HGPS patients compared with the BJ cell line derived from a normal newborn individual (Table S1).

### 2.2 Transient hTERT mRNA expression extends telomeres

**Figure 2:**
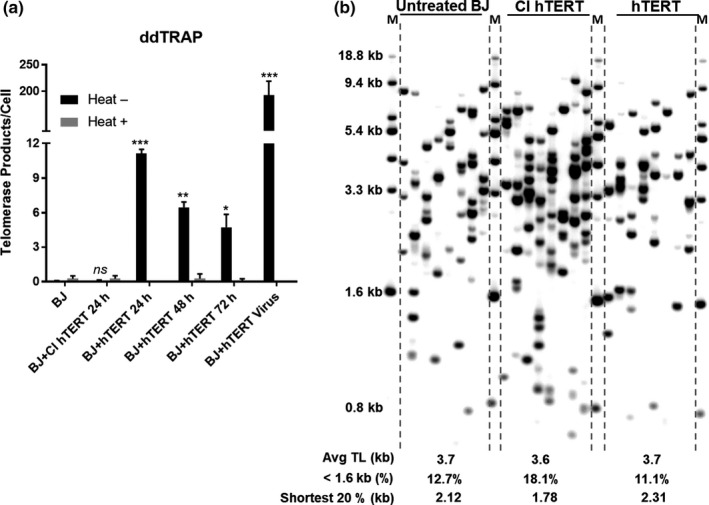
Transient hTERT mRNA expression extends telomeres. (a) Telomerase activity in BJ fibroblasts transfected hTERT or CI hTERT mRNA (1 µg/ml) or hTERT retrovirus, as measured by ddTRAP. *p < 0.05;**p < 0.01;***p < 0.001; ns, not significant from untreated sample (Student's paired *t* test). “Heat +” indicates the samples were heated to inactivate telomerase. (b) Short telomere distribution in untreated BJ (PD55), hTERT, or CI hTERT mRNA consecutively treated BJ (every 48 hr for four times), as detected by telomere shortest length assay

A previous report indicated constitutively expressing telomerase was able to increase the proliferation capacity of HGPS cells (Benson, Lee, & Aaronson, 2010; Kudlow, Stanfel, Burtner, Johnston, & Kennedy, 2008). To avoid insertional mutagenesis and cell immortalization, we decided to determine whether transient expression of telomerase could at least partially rescue the progeria telomere defects. We measured telomerase activity after normal human fibroblasts BJ were transfected with hTERT or catalytically inactive (CI) hTERT mRNA. The CI hTERT has a dominant negative point mutation at one of the triad of metal‐coordinating aspartates at the catalytic site and abolishes telomerase activity (Wyatt, 2009). Using hTERT mRNA, telomerase activity peaked at 24 hr after a single transfection and was maintained for 3 days. We did not detect telomerase activity in CI hTERT mRNA‐treated cells. Compared to transfecting an hTERT cDNA‐containing retrovirus, hTERT mRNA did not confer strong telomerase activity (Figure 2a and Figure S3).

Transient introduction of hTERT mRNA into cells lengthened the shortest telomeres, indicating the telomerase activity produced was functional on telomeres. We transfected BJ cells (PD55) with hTERT or CI hTERT mRNA four times in succession at 48‐hr intervals and then performed TeSLA. There was not a significant increase in the average telomere length in the hTERT mRNA‐expressing cells, but there was extension of short telomeres (20% shortest telomeres: 2.12 kb in untreated; 2.3 kb in hTERT mRNA‐treated cells). After hTERT RNA introduction, the percentage of the shortest telomeres (those below 1.6 kb) was modestly reduced (12.7% in untreated cells; 11.1% in hTERT mRNA‐treated cells). CI hTERT did not extend short telomeres (Figure 2b). We observed the same results in hTERT mRNA‐treated HGPS cells (Figure S4). Thus, transient introduction of hTERT mRNA may elongate the short telomere potentially preventing cell cycle arrest and senescence.

### 2.3 Telomerase mRNA transient expression increases the replicative capacity of HGPS cells with short telomeres

**Figure 3:**
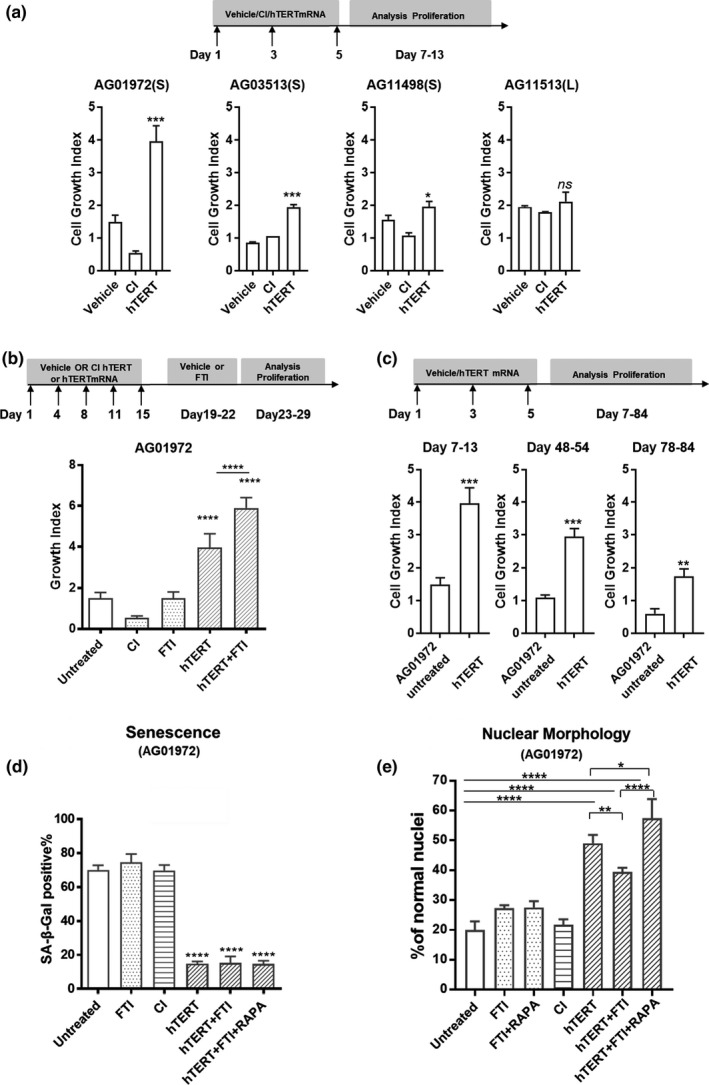
Transient hTERT mRNA expression stimulates proliferation of short telomere progeria cells and reversed aging of progeria cells. (a) Proliferation curves were generated by *xCELLigence*. Cells at a seeding density of 5,000/ well were monitored during 7‐day incubation. All histograms represent results from three independent experiments ± *SD*;***p < 0.001; **p < 0.01. (b) Lonafarnib and hTERT transient expression synergistically enhanced Hutchinson–Gilford progeria syndrome cell proliferation. ****p < 0.0001. (c) AG01972 were treated with hTERT mRNA (1 µg/ml) every 48 hr for three times. Proliferation data were generated by *xCELLigence*. (d) Quantification of β‐gal positive staining AG01972 that were untreated; or exposed to lonafarnib (FTI, 1 μM) for 2 weeks; or transfected (3× at 48 hr intervals with CI or hTERT mRNA); or exposed to the combination of hTERT transfection, followed by 2 weeks of treatment with lonafarnib or everolimus (RAPA; 10 nM). ~100 cells per sample were scored manually. Error bar indicates *SD*; *n* = 100; ****p < 0.0001. (e) Quantification of nuclear morphology. AG01972 (P21) were treated with hTERT or CI hTERT mRNA (1 µg/ml) every 48 hr for three times. Cells were cultured 2 weeks, followed by 2 weeks of treatment with lonafarnib (FTI, 1 μM) or in combination with everolimus (RAPA; 10 nM). Nuclei were stained using antilamin A/C. Bars show the mean frequency of normal shaped nuclei from two independent experiments. The data were calculated over 300 nuclei per condition, in a double‐blinded fashion. Error bar indicates *SD*;*p < 0.05; **p < 0.01; ****p < 0.0001

We next tested if transient expression of telomerase mRNA provided extended proliferation potentially providing benefits to HGPS patient cells. Three different HGPS cell lines (AG01972; AG03513; and AG11498) characterized by short telomeres were examined as well as one HGPS cell line (AG11513) that had normal telomere length. We transfected each of these cells with hTERT or CI hTERT mRNA three times in succession at 48‐hr intervals, and after the last transfection monitored real‐time proliferation. In each of the HGPS cell lines characterized as short telomeres, proliferative capacity increased after hTERT mRNA treatment (Figure 3a; Figure S5A,B). In contrast, the replicative capacity of cells derived from AG11513, where telomere length was not short, was unaffected by hTERT mRNA treatment (Figure 3a; Figure S5A). Thus, progeria patient cells with short telomeres, but not those with normal telomere lengths, may respond differently to transient telomerase therapy. In contrast, CI hTERT‐treated cells did not increase cell proliferation in any of the patient samples, confirming that the activity of telomerase to extend telomeres (as opposed to other telomerase‐related functions) is necessary for the enhancement of replicative capacity (Figure 3a). It is well established that telomerase preferentially elongates the shortest telomeres (Steinert, Shay, & Wright, 2000). In contrast to introducing transient TERT mRNA, we did not observe a benefit of lonafarnib on cell replication (Figure 3b; Figure S6). However, when we combined lonafarnib treatment with hTERT mRNA, we observed enhanced cell proliferation compared to either treatment alone (*p* < 0.0001; Figure 3b). This suggests hTERT mRNA and FTIs enhance HGPS cell proliferative capacity by different mechanisms.

### 2.4 Short exposure to hTERT mRNA maintains long‐term proliferation of HGPS cells with short telomeres

Transient expression of telomerase had a long‐term effect on the proliferation of progeria cells, reducing cell loss and extending overall cell proliferation well beyond untreated cells. Progeria cells (AG01972) underwent three consecutive transfections with hTERT mRNA at 48‐hr intervals. After 30 days, untreated progeria cells entered senescence, while hTERT mRNA‐treated cells continued to proliferate even after 3 months (Figure 3c). We observed this long‐term effect on proliferation after transient hTERT mRNA treatment in fibroblasts from other short telomere HGPS patients (Figure S7A). We analyzed short exposure to hTERT mRNA in short telomere BJ (PD 60) cells and observed enhanced cellular proliferation. In contrast, long telomere BJ (PD28) and progeria cells (HGADFN127 and HGADFN003) did not show growth enhancement by hTERT mRNA, even in long‐term culture (Figure S8). These results are similar to previous studies introducing a retroviral construct containing hTERT cDNA flanked by loxP‐sites. Introduction of floxable TERT cDNA into late passage almost senescent BJ cells with very short telomeres followed by excising TERT gene ~2 weeks later resulted in an extension of cell proliferation for an extra 50 population doublings compared with only 5 population doublings for vector control infected BJ cells (Steinert et al., 2000). Since this and other studies (Hemann, Strong, Hao, & Greider, 2001) show that telomerase preferentially elongates the shortest telomeres, which are rate limiting for cell proliferation, this indicates a transient introduction of telomerase may have large effects.

### 2.5 Transient telomerase expression reverses signs of HGPS cellular senescence

#### 2.5.1 Senescence

We observed 70% of AG01972 progeria cells stained for senescence‐associated β‐galactosidase (*SA‐β‐gal*). SA‐*β‐gal* + cells were dramatically reduced (from 70% to 15% SA‐*β‐gal* + cells per field of view) after hTERT mRNA treatment. We did not observe this benefit in cells treated with CI hTERT or lonafarnib (70% or 74% SA‐β‐gal + cells after treatment, respectively). Combining hTERT with lonafarnib (FTI) or lonafarnib and everolimus (Rapamycin, RAPA), each of which has been previously tested in HGPS in clinical trials, did not further reduce the percentage of SA‐*β‐gal* + cells (Figure 3d and Figure S9).

#### 2.5.2 Senescence‐associated secretory phenotype

Senescent cells secrete a variety of inflammatory cytokines, known as the senescence‐associated secretory phenotype (SASP), triggered by persistent DNA damage (Rodier et al., 2009; Tedone et al., 2019). Vascular inflammation, calcification, and the progression of atherosclerosis are also associated with SASP (Csoka et al., 2004). We measured the expression of inflammatory cytokines in progeria (AG03513 and AG11498). In HGPS fibroblasts, the mRNA levels of inflammatory cytokines (IL1A, IL1B, IL6, and IL8), chemokines (CXCL1, CXCL2), and the adhesion molecule ICAM1 were markedly elevated (40–3,600 fold for AG03513, 2–50 fold for AG11498; Figure S10A).

We validated the increased secretion of IL‐6 in progeria cells by ELISA (AG01972, 10‐fold by comparison to WT; Figure S10B). Notably, three consecutive treatments with hTERT mRNA substantially reduced IL‐6 expression (*p* < 0.001). We observed IL‐6 secretion was reduced by 30% and 80% at days 7 and 17 after the treatment with hTERT mRNA. In contrast, CI hTERT did not reduce IL‐6 secretion, suggesting that telomerase catalytic activity was responsible for the resolution of SASP (Figure S10B).

Surprisingly, 2 weeks of lonafarnib (FTI) treatment exacerbated SASP, as reflected by a significant increase in IL‐6 secretion. Co‐administration of hTERT mRNA partially mitigated this adverse effect of lonafarnib. Adding everolimus, which has an anti‐inflammatory function, did not reduce the generation of IL‐6 (Figure S10B). We examined a larger set of inflammatory cytokines in an additional progeria cells (AG03513) after transient expression of hTERT and found reduced mRNA levels of IL6, IL8, IL1B, and GM‐CSF (Figure S5C–I). We confirmed the maintenance of long‐term proliferation, an increase in cell number, and the reversal of senescence in this additional patient. (Figure S5C–I).

#### 2.5.3 Nuclear morphology

Hutchinson–Gilford progeria syndrome cells exhibit blebbing of the nuclear membrane, due to the accumulation of progerin at the nuclear envelope. Treatment with either FTIs or rapamycin partially normalizes nuclear dysmorphism (Cao et al., 2011; Verstraeten, Ji, Cummings, Lee, & Lammerding, 2008). Accordingly, we determined whether transient telomerase expression alone or in combination with FTI and/or rapamycin could restore nuclear morphology. HGPS cells (AG01972) underwent three consecutive transfections with hTERT or CI hTERT mRNA, followed by lonafarnib (FTI; 1 µM) and/or everolimus (Rapamycin; 10 nM) for 2 weeks, and were stained with lamin A/C antibody to visualize nuclear membrane. Analysis of ~300 cells for each condition revealed that without treatment HGPS cells had extensive nuclear blebbing and wrinkles (only 20% of nuclei had normal morphology; Figure 3e; Figure S11A). Treatment with lonafarnib (1 µM) alone or with everolimus (10 nM) improved nuclear shape as previously reported (Capell et al., 2005; Gordon et al., 2012) to ~27% having normal nuclei; Figure 3e; Figure S11C). Unexpectedly, hTERT treatment was superior to lonafarnib alone or everolimus (with 49% of cells having a normal nuclear morphology after hTERT treatment; Figure 3e; Figure S11D). Because CI hTERT did not improve the nuclear shape (22% of cells exhibiting normal nuclei; Figure 3e; Figure S11B), the benefit of hTERT is likely due to telomere extension. The combination of hTERT with lonafarnib did not further improve nuclear morphology (Figure 3e; Figure S11E; 39% of cells with normal nuclei respectively; *p *< 0.0001). The combination of hTERT with lonafarnib and everolimus tended to further improve nuclear morphology (58% of cells with normal nuclei; *p* < 0.0001; Figure 3e; Figure S11F). We also observed a worsening of nuclear blebbing with increased passage number, as previously reported (Goldman et al., 2004). As telomere length shortens with passage number, this observation is also consistent with an interaction between telomere length and nuclear morphology in progeria.

### 2.6 Transient transfection with hTERT mRNA partially rescues progerin‐interactive telomere‐associated proteins

**Figure 4:**
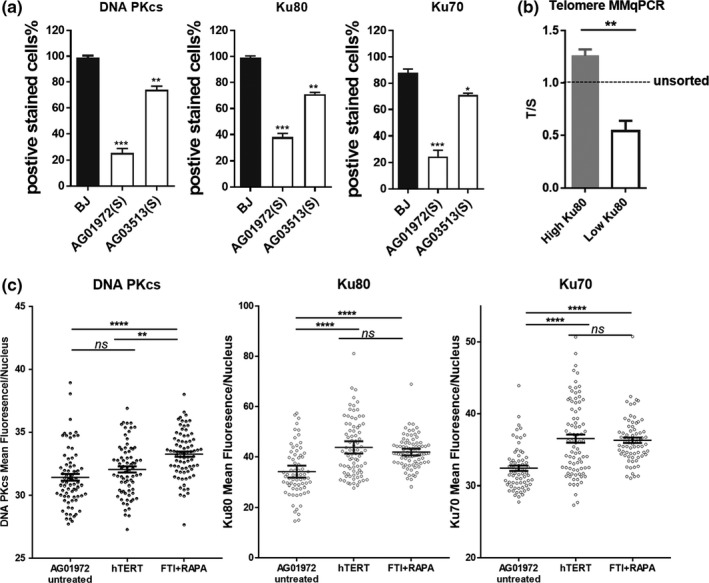
Transient expression of hTERT mRNA restores progerin‐interactive telomere binding proteins DNA PKcs/Ku70/Ku80 loading on telomeres. (a) Fibroblast cells were stained by anti‐DNA PKcs, anti‐Ku80, and anti‐Ku70 antibodies and DAPI. Error bar indicates *SD*; *n* = 200 nucleus; *p <0.05; **p <0.01; ***p < 0.001. (b) 2 × 10^6^ AG03513 and BJ cells were stained with Ku80‐AF647 antibody and then sorted into high‐ and low‐signal Ku80 cells by FACS. Average telomere lengths were analyzed by Telomere MMqPCR. Error bar indicates *SD*; *n* = 3; **p < 0.01. (c) AG01972 (P21) were treated with hTERT or CI hTERT mRNA (1 µg/ml) every 48 hr for 3 times. Cells were cultured 1 week, followed by 1 week of treatment with lonafarnib (FTI, 1 μM) or in combination with everolimus (RAPA; 10 nM). Cells were fixed and stained by anti‐DNA PKcs, anti‐Ku80, and anti‐Ku70 antibodies. Mean fluorescence intensity of each nucleus was measured by Image J. Error bar indicates *SEM*; *n* = 80; **p < 0.01;****p < 0.0001; ns, not significant

A previous report indicated progerin is a binding partner of lamin B1/B2, emerin, and DNA PKcs. Furthermore, ectopic expression of progerin reduces the expression of DNA PKcs (Liu et al., 2011). DNA PKcs and its regulatory subunits Ku70 and Ku80 are telomere‐associated proteins that form complexes regulating telomere length (Myung et al., 2004; Ruis, Fattah, & Hendrickson, 2008; Wang, Ghosh, & Hendrickson, 2009). We confirmed a significant reduction in the expression of nuclear DNA PKcs, Ku70, and Ku80 in progeria fibroblasts (AG01972) when compared to BJ (*p* < 0.0001; Figure 4a; Figure S12A).

From the same patient sample, we observed heterogeneity in the cellular expression of the telomere binding proteins. To determine if there might be a correlation between expression of these telomere binding proteins and telomere length, we separated HGPS cells with high Ku80 expression from cells with low expression using FACS. HGPS cells with preserved Ku80 expression had significantly longer telomeres (*p* < 0.01; AG03513; Figure 4b).

We then examined if transient expression of telomerase can rescue the expression of the telomere binding proteins DNA PKcs, Ku70, and Ku80. We compared the effect of hTERT alone to that of the lonafarnib (1 µM) combined with everolimus (10 nM) (Figure 4c; Figure S12B). Lonafarnib reduces farnesylation of progerin, while everolimus reduces progerin levels by promoting autophagy (Cao et al., 2011). For each treatment, we analyzed ~80 nuclei. After 1 week of treatment, we observed lonafarnib (FTI) (1 µM) combined with everolimus (RAPA) (10 nM) restored the expression of DNA PKcs, Ku70, and Ku80 in progeria cell nuclei. In comparison, transient expression of telomerase mRNA partially rescued Ku70 and Ku80 but had minimal effects on DNA PKcs (Figure 4c; Figure S12B). Thus, hTERT mRNA transfection can partially restore DNA PKcs/Ku70/Ku80 in progeria cells.

### 2.7 Transient expression of hTERT mRNA does not rescue epigenetics changes, which are associated with telomere loss

**Figure 5:**
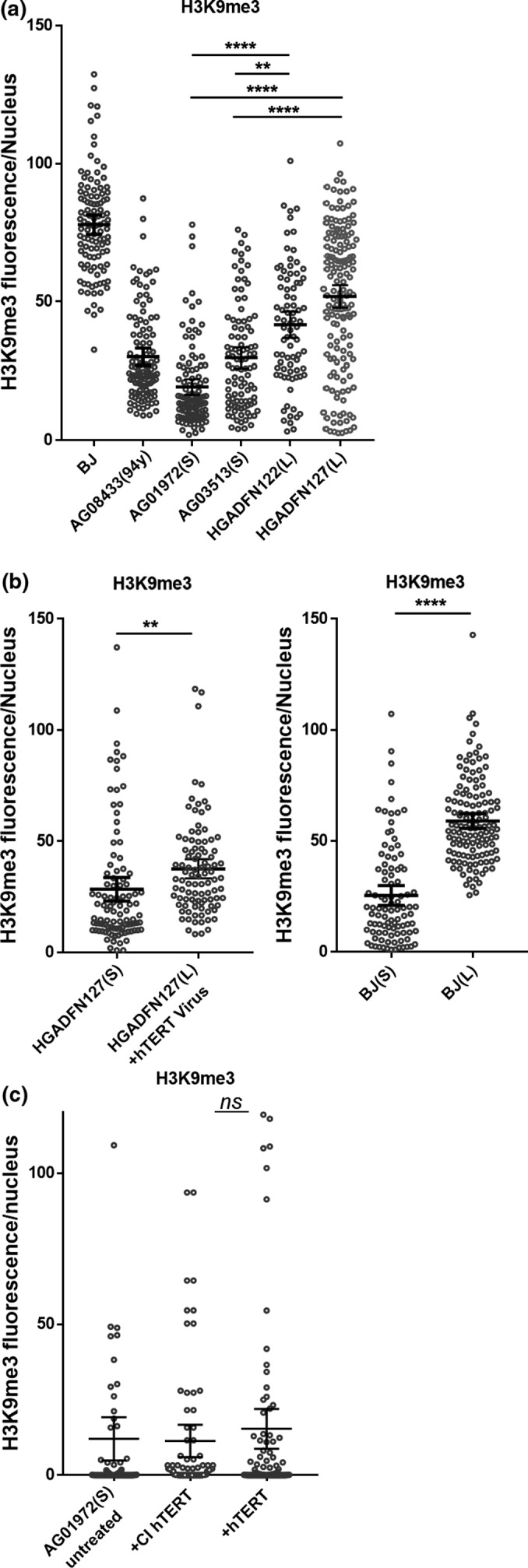
Loss of heterochromatin in progeria cells is associated with telomere shortening. (a) Fibroblast cells were stained by anti‐H3K9me3. H3K9me3 fluorescence intensity for each nucleus was plotted. Error bar indicates *SD*; *n* = 100; ****p < 0.0001. **p < 0.01. (b) BJ or HGADFN127 cells from isogenic clones with short and long telomeres were stained by anti‐H3K9me3. Error bar indicates *SD*; *n* = 3; ****p < 0.0001. **p < 0.01. (C) AG01972 were treated with hTERT or CI hTERT mRNA (1 µg/ml) every 48 hr for three times. Cells were stained 14 days after the last treatment by anti‐H3K9me3. Error bar indicates *SD*; *n* = 100; ns, not significant

Profound changes in histone modifications of HGPS cells have been widely reported (Shumaker et al., 2006). Normally, nuclear lamina is associated with heterochromatin, marked by H3K9me3 and the associated protein HP1. In HGPS, peripheral heterochromatin and the relative histone modifications are lost (Shumaker et al., 2006). We examined the heterochromatin marker H3K9me3 in progeria cells and found H3K9me3 decline was associated with telomere loss. We analyzed ~100 nuclei per patient cell strain. The average H3K9me3 intensity was lower in short telomere progeria cells compared with long telomere cells (*p* < 0.01 for AG03513; *p* < 0.0001 for AG01972; Figure 5a; Figure S13A). Compared to BJ fibroblasts, H3K9me3 was significantly reduced in long telomere progeria cells, indicating progerin is important in global epigenetic changes. The loss of H3K9me3 was also observed in normal aging. A 94‐year‐old healthy subject (AG08433) had significant lower H3K9me3 compared with BJ (*p* < 0.0001; Figure 5a; Figure S13A), suggesting H3K9me3 may be a biomarker for normal aging.

We then tested if elongation of telomeres can rescue the epigenetic changes in progeria. We employed isogenic cell lines with long and short telomere BJ cells, which were generated by transduction with a floxed hTERT, yielding isogenic subclones with experimentally manipulated telomere length (long 11 kb and short 5 kb by TRF; Robin et al., 2014). Elongation of telomeres in BJ significantly increased levels of H3K9me3 (*p* < 0.0001; Figure 5b and Figure S13B). We generated long and short telomere progeria cells from patient (HGADFN127) by retroviral transduction of telomerase, while the uninfected progeria cells were passaged for additional 30 PDs. Long telomere progeria cells maintained H3K9me3 in nearly every nucleus, while the isogenic matched cells with short telomeres lost H3K9me3 (Figure 5b; Figure S13B–C).

We then examined if transient exposure of hTERT mRNA can rescue the epigenetic changes in progeria cells. Progeria cells (AG01972) were treated with three consecutive transfections with hTERT or CI hTERT mRNA in succession at 48‐hr intervals. H3K9me3 was examined 2 weeks after the last treatment. The level of H3K9me3 was not increased by hTERT mRNA (Figure 5c and Figure S13D), suggesting the partial rescue of epigenetic changes in progeria requires longer‐term expression of telomerase in cells and perhaps more significant elongation of telomeres.

## 3 DISCUSSION

### 3.1 Telomere shortening in HGPS

Most HGPS fibroblasts demonstrate rapid telomere erosion and senescence (Scaffidi & Misteli, 2008). The mean telomere lengths are reduced in the majority of HGPS patients (Figure S1). The decreased telomere length may be caused by a large amount of telomeric DNA lost with each cell division or an increase in apoptosis in HGPS cells leading to an increase in divisions in the remaining cells. It was reported the telomere shortening rate is increased in some HGPS patients in certain chromosomes (Turner, 2014).There is a 4‐ to 8‐fold increase in the rate of apoptosis in HGPS fibroblasts compared with normal fibroblasts during cellular aging (Bridger & Kill, 2004). It is possible that the remaining progeria cells undergo successive rounds of cell division to compensate for the gradual cell loss. When telomeres are critically short, DNA damage signaling is triggered and the cell cycle is stalled. Therefore, progeria cells with very short telomeres gradually accumulate and become senescent. Our TeSLA verified not only average telomere lengths are shorter in most HGPS patients, but also the percentage of the shortest telomeres is higher compared with wild‐type cells (Figure 1). The heterogeneity of telomere length in cell populations of the same progeria individual is supported by our observation that the intra‐individual cell heterogeneity of expression of telomere binding proteins DNA PKcs/Ku70/Ku80 directly correlates with low‐expressing cells having shorter telomeres (Figure 4).

Hutchinson–Gilford progeria syndrome fibroblasts demonstrate loss of heterochromatin. H3K9me3 is one of the constitutive heterochromatin markers of the pericentric region and telomeres (Shumaker et al., 2006). Progerin decreases H3K9me3 levels globally. Although progerin is the major factor affecting H3K9me3, telomere shortening also regulates the epigenetic status of chromatin. Long telomere progeria cells preserve higher H3K9me3 compared with short telomere cells. Moreover, with telomere progressive shortening, H3K9me3 declines further (Figure 5). This is consistent with a previous study using the Terc−/− mice with short telomeres showing decreased H3K9me3 in telomeric and subtelomeric chromatin (Benetti, Garcia‐Cao, & Blasco, 2007). Elongation of telomeres both in BJ and progeria cells by constitutively expressing telomerase rescues H3K9me3 (Figure 5).

### 3.2 Transient hTERT mRNA exposure reverses senescence of HGPS

Telomerase can prevent progerin‐induced proliferation inhibition. Exogenous progerin impairs cell proliferation in primary human fibroblasts. Inactivation of p53 rescues the growth of progerin‐expressing cells. Interestingly, TERT immortalized cells exposed to progerin immediately after hTERT expression shows no change in growth rate (Kudlow et al., 2008). Considering these cells may already grow an additional ~10 PDs during immortalization by hTERT virus and clonal outgrowth assay, their telomeres are extended. The protection effect of telomerase might result from telomere extension or blocking the signals that lead to growth arrest to bypass p53 activation‐induced senescence.

Sustained telomerase expression raises a safety concern of insertional mutagenesis and the increased cancer consequences of long‐term immortalizing cells. Our approach does not transform cells. As expected even with transient expression of hTERT mRNA into short telomere progeria cells (Figure S5B), the cells grew substantially longer but also continued to slowly shorten the telomere length. After the last liposomal transfection with hTERT mRNA, telomerase activity returns to basal levels by 72 hr (Figure 2; Figure S3). We tested for HGPS senescent phenotypes to assess the effect of hTERT mRNA. Three consecutive transfections of hTERT mRNA selectively extend the shortest telomeres, whereas CI hTERT does not (Figure 2; Figure S4). HGPS cell lines having short telomere lengths respond to hTERT mRNA introduction and exhibit an increase in cell proliferation (Figure 3 and Figure S5B). Furthermore, hTERT treatment for these HGPS cells reverses signs of senescence (as manifested by reduced β‐galactosidase expression and the reduced generation of inflammatory cytokines). One possible explanation for these results is that a subset of cells died after consecutive hTERT mRNA treatments due to lipofectamine toxicity, and we selectively monitored the cells that survived by activation of telomerase. However, this did not occur using the catalytically inactive (CI) form of hTERT mRNA so selective toxicity is unlikely. In addition, transient telomerase expression restored nuclear morphology of HGPS cells (Figure 3). Conversely, none of these improvements in phenotypes and functions was observed after treatment with the catalytically inactive (CI) form of hTERT, indicating telomere extension is contributing to these biological responses. These observations support the notion that telomere erosion plays a critical role in the pathophysiology of HGPS, and even transient expression of hTERT mRNA is sufficient to extend cell proliferation.

**Figure 6:**
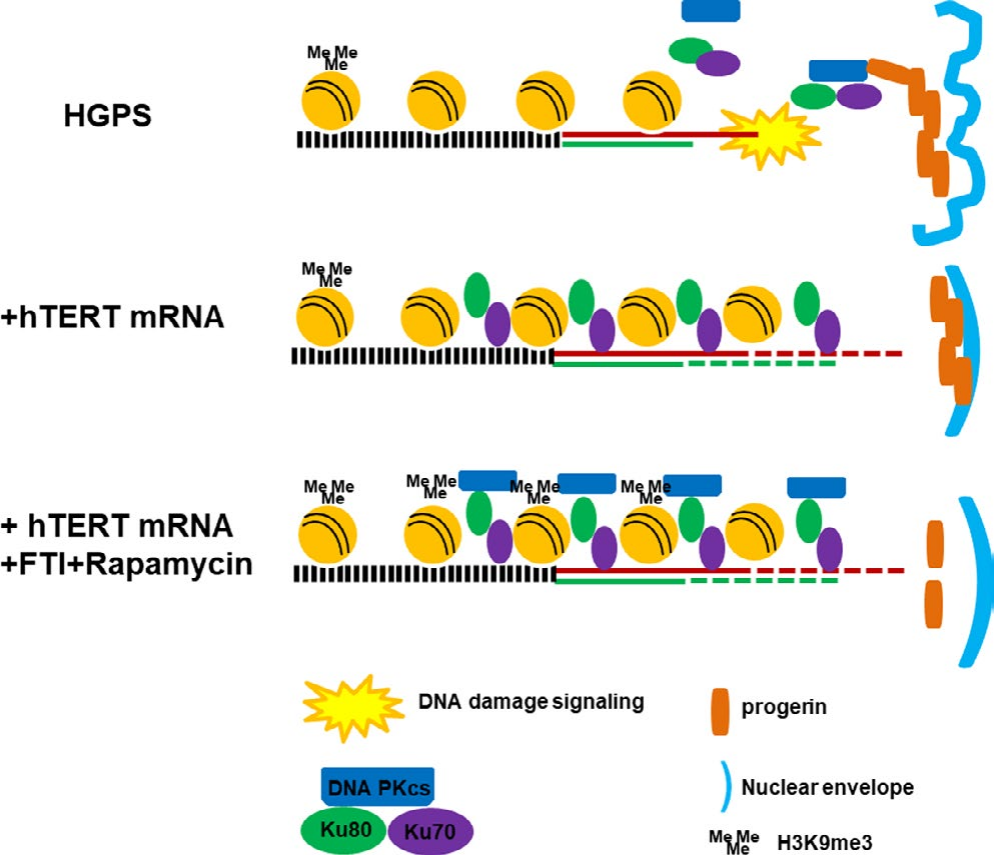
Model for connecting progerin and telomeres in Hutchinson–Gilford progeria syndrome (HGPS). Progerin accumulates at the nuclear inner membrane, interferes with DNA PKcs/Ku70/Ku80 complex formation, and accelerates telomere loss. Short and/or unprotected telomeres in HGPS cells trigger DNA damage signals, resulting in arrested cell cycle, SASP, reduced proliferation, nuclear dysmorphism, and loss of heterochromatin. Transfection with hTERT mRNA increases telomerase activity, extends the shortest telomeres, and promotes Ku70/Ku80 binding. Further decreasing progerin level by FTI and rapamycin restores DNA PKcs. These changes together ameliorate many aspects of the HGPS cell phenotype

In addition, combination of hTERT mRNA and FTIs further improves cell proliferation. FTI‐induced cytokine expression in progeria cells is reduced in hTERT mRNA‐expressing cells (Figure 3). Transient expression of hTERT mRNA alone partially rescues telomere binding protein DNA PKcs/Ku70/Ku80. In combination treatment with FTIs to reduce progerin accumulation, the complex is largely rescued (Figure 4; Figure 6). Thus, progeria cell telomeres are not only extended by hTERT mRNA, but they may be stabilized by the restoration of the telomere binding protein complex. These results indicate telomerase and FTI target distinct pathways.

In summary, although progerin accumulation in cells is the cause of HGPS, telomere shortening exaggerates and worsens the phenotypes. Transient expression of hTERT mRNA rapidly restores almost all functions to HGPS cells without transforming cells. Telomere rapid erosion in progeria cells is clearly secondary to progerin toxicity, and thus, HGPS has been termed a secondary telomeropathy (Opresko & Shay, 2017). However, lamins are scaffolds for genome organization and telomeres/telomere binding proteins, and nuclear lamins are physically and functionally connected (Burla, Torre, & Saggio, 2016; Chojnowski et al., 2015). These new results suggest a possible direct impact of progerin on telomere localization, structure, and function and may lead to new translational opportunities.

## 4 MATERIAL AND METHODS

### 4.1 hTERT mRNA

hTERT and CI hTERT mRNAs were provided by the RNACore of Houston Methodist Research Institute and phaRNA, LLC. The ORF of hTERT is human TERT transcript variant 1 (NM_198253.2), and the CI TERT has a point mutation D712A (Ramunas et al., 2015). mRNAs were generated by in vitro transcription and modified with pseudouridine and 5‐methylcytidine to increase stability, followed by HPLC purification to remove abortive transcripts and RNA‐DNA template hybrids.

### 4.2 Retroviral infection

The construction of vector (hTERT‐pBabe‐Hygro) and stable retrovirus‐producing cell line was described previously (Zhu et al., 2007). Fibroblast cells were infected with retroviral supernatant containing 4 μg/ml polybrene (Millipore). Selection was started 48 hr after infection by 200 μg/ml hygromycin (Invitrogen) for 1 week.

### 4.3 Droplet digital TRAP assay

Telomerase activity assay was performed as described (Ludlow et al., 2014). Cells were lysed in NP‐40 buffer on ice for 45 min and added to the telomerase extension reaction for 40 min. Telomerase was heat inactivated. An aliquot of the extension products was amplified by droplet PCR, and fluorescence was measured on the droplet reader (Bio‐Rad).

### 4.4 TRF analysis

Genomic DNA was extracted by Gentra Puregen DNA Extraction Kit (Qiagen). DNA (3 μg) was digested with HinF I and Rsa I (NEB) overnight and separated on a 0.85% agarose gel at 2 V/cm for 16 hr. DNA was transferred from gel to Hybond‐N+ membrane (GE) and fixed by UV crosslinking. The membrane was hybridized with DIG‐labeled telomere probe overnight at 42 °C, followed by washing with 2× SSC, 0.1% SDS buffer for 15 min, 0.5× SSC, and 0.1% SDS buffer at 55 °C for 15 min twice. The membrane was incubated with 1× DIG blocking solution and then with anti‐DIG antibody (Roche) for 30 min. Telomere signals were detected by incubating with CDP‐star (Roche) for 5 min. The average telomere length was quantified using telotool software.

### 4.5 Telomere shortest length assay

Telomere shortest length assay was performed as described (Lai et al., 2017). Genomic DNA was ligated to telomere linkers by T4 DNA ligase (NEB), followed by digestion with a mixture of restriction enzymes CviAII, BfaI, NdeI, and MseI (NEB). DNA was treated with Shrimp Alkaline Phosphatase (NEB) followed by adaptor ligation. 1 μM of AT and TA adapter was ligated to the DNA fragments by T4 DNA ligase. Telomeres were amplified by PCR (94°C for 2 min, 26 cycles of 94°C for 15 s, 60°C for 30 s, and 72°C for 15 min) and resolved on a 0.85% agarose gel (1.5 V/cm for 21 hr). Southern blot was performed as in TRF analysis to detect telomeres.

### 4.6 Telomere‐FISH

Cells were treated with colcemid (6–12 hr at 0.1 mg/ml) before exposed to 75 mM KCl for 5 min at 37°C, then resuspended in fixative, and dropped onto a clean slide. FISH was performed with the Telomere PNA Kit (Dako).

### 4.7 Cell treatment

Primary human fibroblasts were cultured in DMEM (Invitrogen) containing 2 mM glutamax, 0.1 mM nonessential amino acids, and 15% FBS. Cells were transfected with mRNA using Lipofectamine RNAiMax in Opti‐MEM (Life Technologies) at 1 μg/ml for 4 hr. Cells were replenished with fresh medium containing lonafarnib (1 µM; Sigma; Medchem Express) or everolimus (10 nM; Sigma) every 48 hr.

### 4.8 Cell proliferation assay

Real‐time cell proliferation was monitored by *xCELLigence* (Roche). Cells (3–5 × 10^3^) were seeded into the 16‐well E‐plates in triplicate. Microelectrodes in the wells provided impedance‐based detection of cellular proliferation. The impedance value of each well was expressed as Cell Index value.

### 4.9 Immunofluorescence and quantification

Cells were fixed with 4% formaldehyde for 15 min, followed by permeabilization in 100% acetone for 10 min at −20°C. After blocking with 4% normal donkey serum for 1 hr, cells were incubated at 4°C overnight with the primary antibody and RT for 1 hr with secondary antibody. Nuclei were stained with DAPI (Life Technologies). Antibodies were anti‐DNAPKcs (ab1832); anti‐Ku70 (ab3114); anti‐Ku80 (sc‐5280); antilamin A/C (sc‐6215); anti‐H3K9me3 (ab8898); Alexa 488‐conjugated goat anti‐mouse IgG (4408S; Cell Signaling); and Alexa 546‐conjugated donkey anti‐goat IgG (A11056; Life Technologies). Images were processed with Image J. H3K9me3 fluorescent images were processed with matlab.

### 4.10 ELISA

Cell culture media were collected from triplicates of each condition. ELISA was performed using kit and procedures from R&D (#D06050). Secretion of IL‐6 was normalized to cell number, reported as pg protein per 10^6^ cells per 24 hr, and plotted as fold changes in comparison with the control cells.

### 4.11 Statistical analysis

Statistical analyses were performed using graphpad prism. Results are presented as mean ± *SEM*/*SD*/95% CI. Comparisons were performed with chi‐squared, Student's *t* test, or one‐way ANOVA. *p* < 0.05 was defined as statistically significant.

## CONFLICT OF INTEREST

None declared.
