# Supplementary material for: Transient introduction of human telomerase mRNA improves hallmarks of progeria cells
Source: Aging Cell. 2019 May 31;18(4):e12979. doi: 10.1111/acel.12979 (PMC6612639; doi:10.1111/acel.12979)
Supplement: Supplementary file 1 [file ACEL-18-e12979-s001.docx]

**Supplemental Figures and Legends**


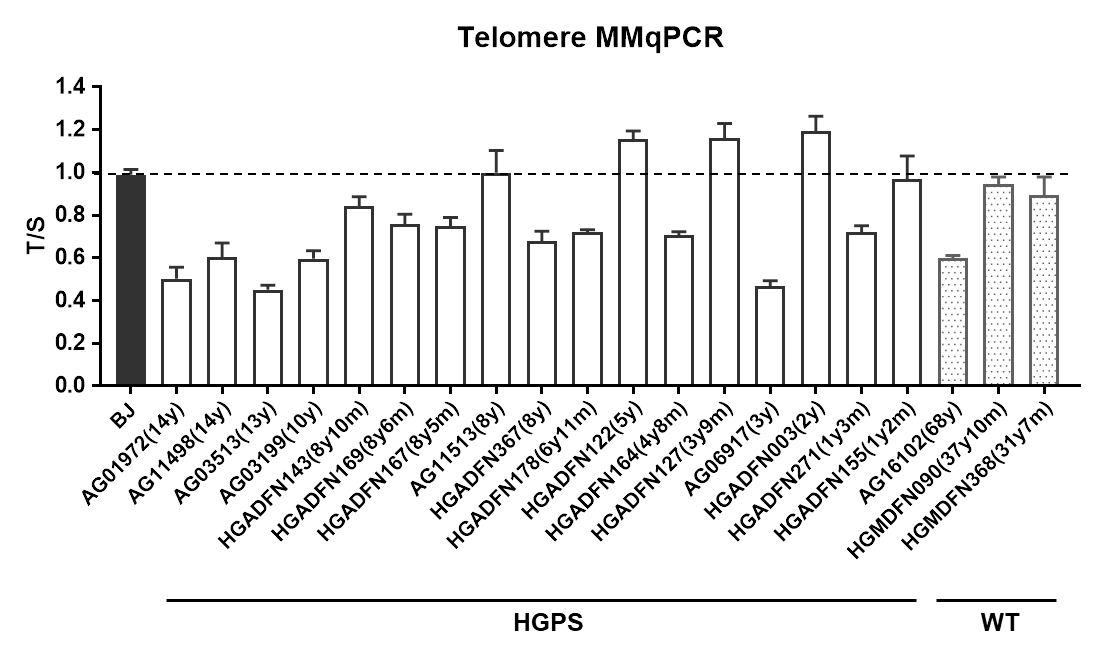


**Supplemental F1.** **Heterogeneity of telomere length in HGPS patients.** Average telomere lengths of 17 HGPS patients and 4 wild-type fibroblasts were analyzed by Telomere MMqPCR. *n=5*.


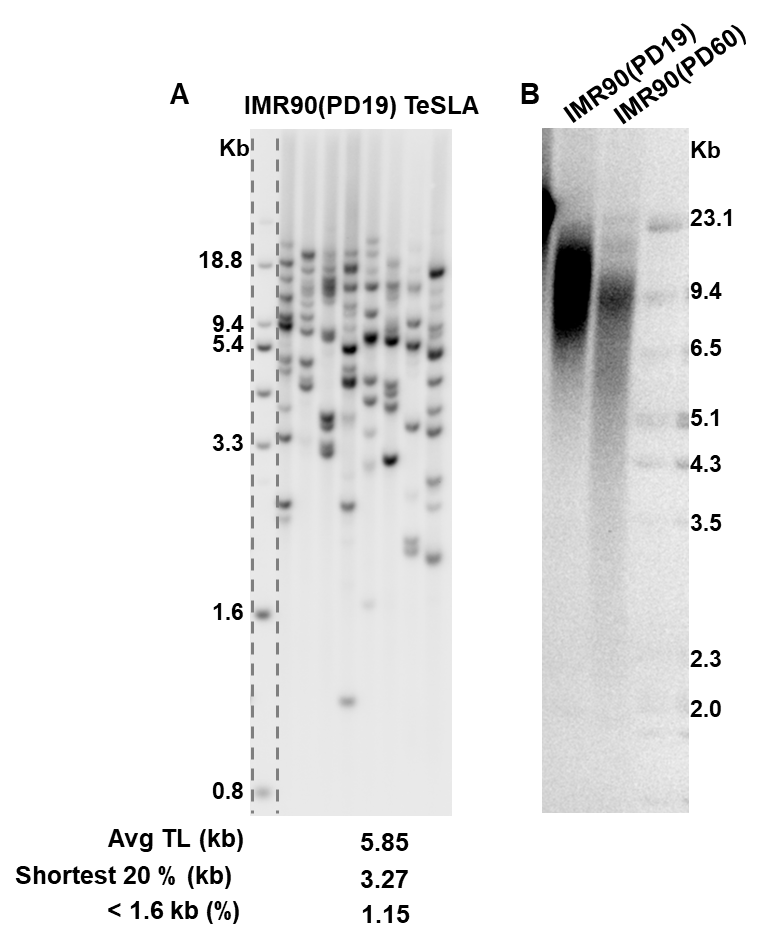


**Supplemental F2. Telomere length and short telomere distribution of an additional normal fibroblast.**  (A) IMR90 (human lung fibroblast) TeSLA. (B) TRF assay.


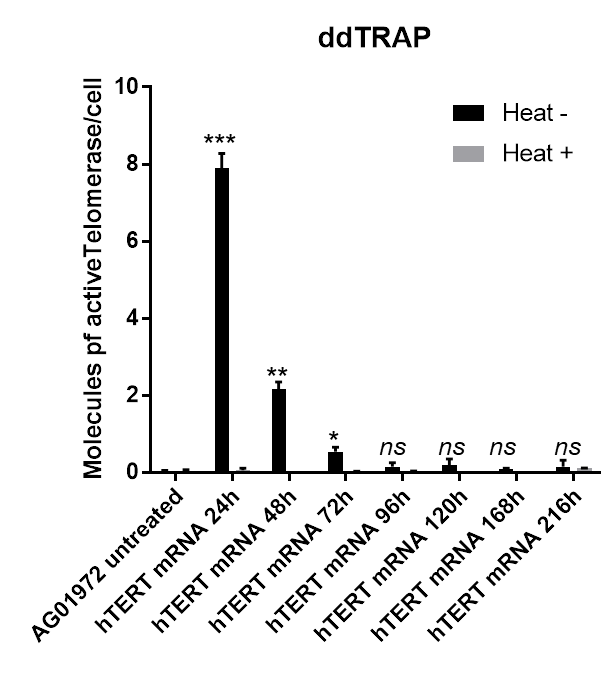


**Supplemental F3. Telomerase activity in hTERT mRNA treated progeria cells.** Telomerase activity in AG01972 cells with a single transfection of hTERT or CI hTERT mRNA (1 µg/ml), measured by ddTRAP. *n=3*. **P < 0.05*;***P <0.01*;****P <0.001*; *ns*, not significant from untreated sample (Student’s Paired t-test).


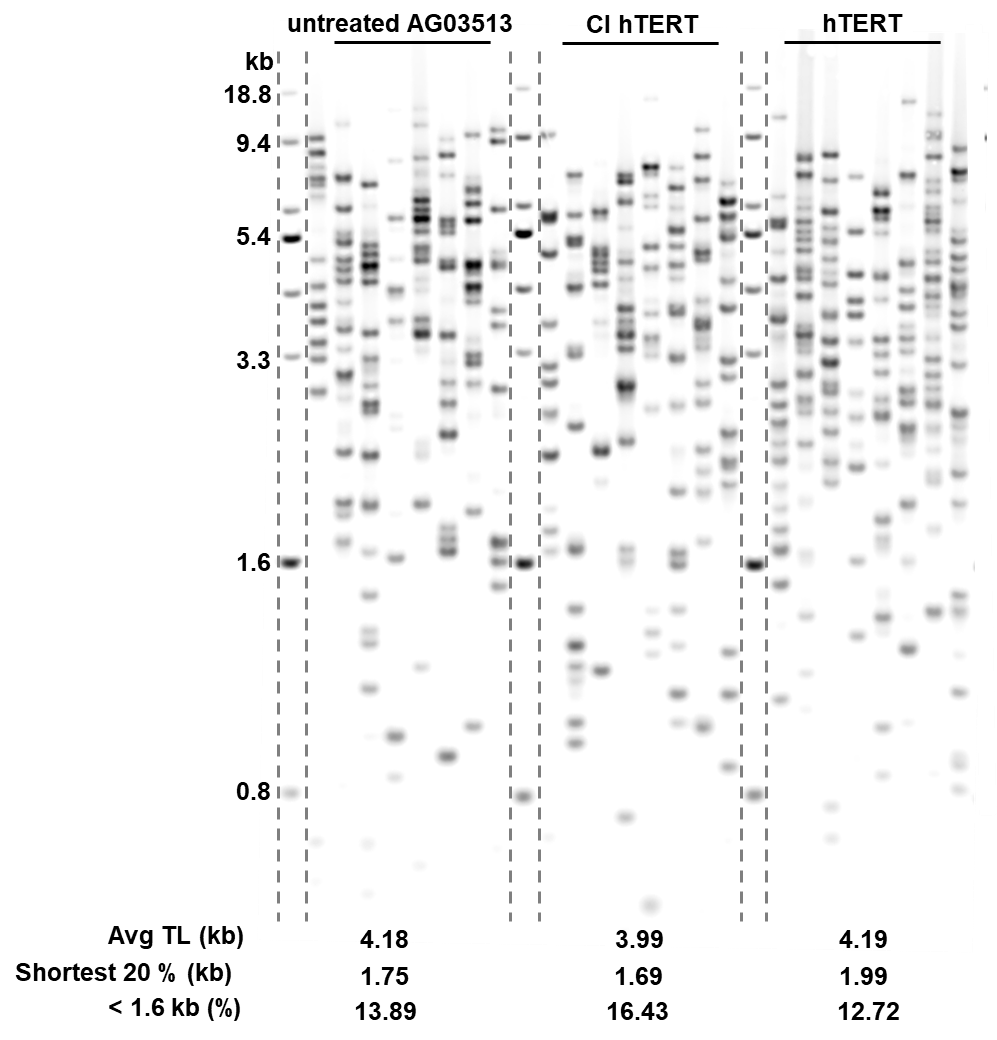


**Supplemental F4. Transient hTERT mRNA expression extends progeria cell telomeres.** Short telomere distribution in untreated, hTERT or CI hTERT mRNA treated progeria cells AG03513 (every 48 hours for 3 times), as detected by TeSLA.


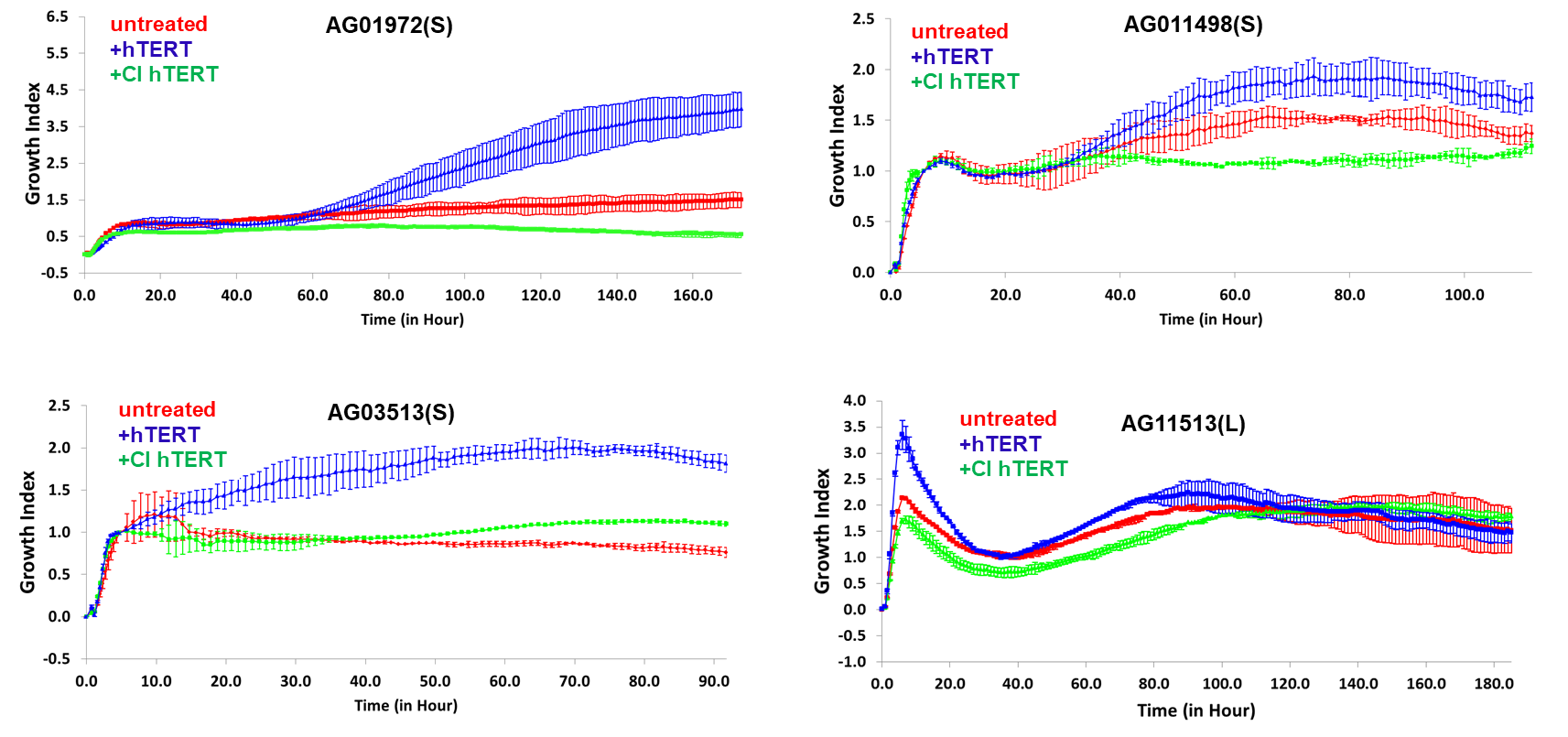


**Supplemental F5A. Proliferation of HGPS cells after hTERT treatment.** Proliferation curves were generated by *xCELLigence*. All graphs represent results from three independent experiments ± SD.


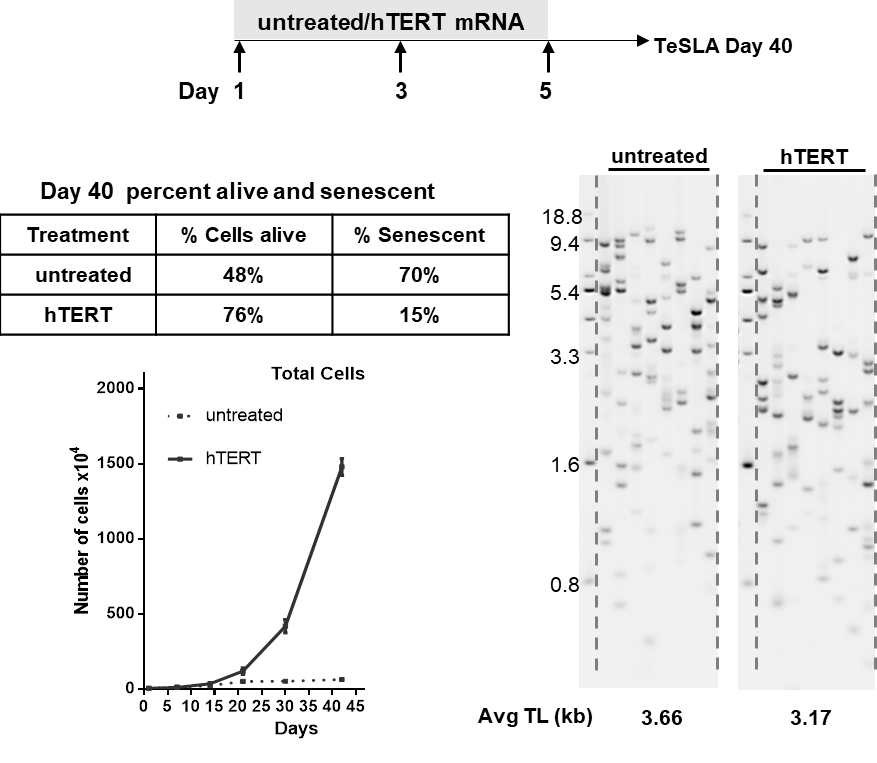


**Supplement F5B.** **Transient hTERT mRNA extends the proliferative lifespan of progeric cells without immortalizing them.** AG01972 progeria cells (P20) have short telomeres and only divide a few times and then undergo senescence or die. At this time the average telomere length is 3.66 Kb. The hTERT mRNA treated cells continue to divide but since this is a transient expression of telomerase it was expected that telomeres might progressively shorten and this is observed at day 40 when the cells had gone through at least 9 additional population doublings compared to the controls. Thus while transient telomerase expression extends the proliferative lifespan of the cells it does not immortalize them and fully maintain telomeres as occurs in cancer cells.


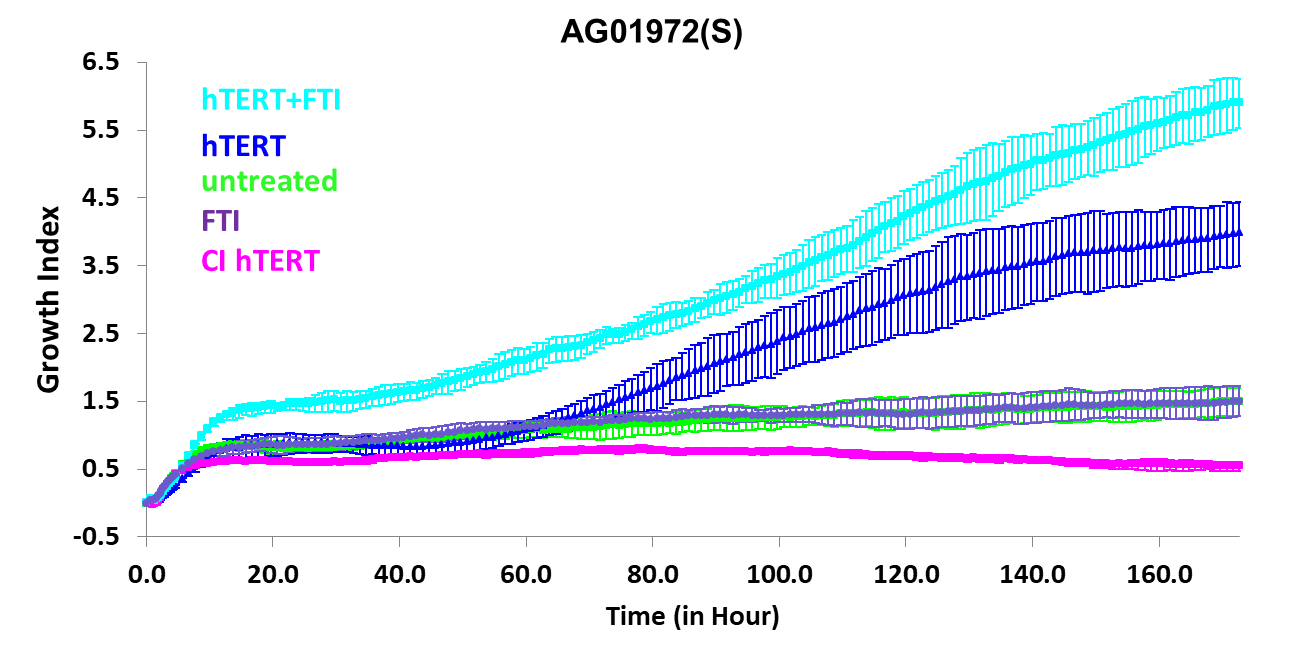


**Supplemental F6. FTI and hTERT transient expression synergistically enhance HGPS cell proliferation.** Proliferation curves of AG01972 were generated by *xCELLigence*. All graphs represent results from three independent experiments ± SD.


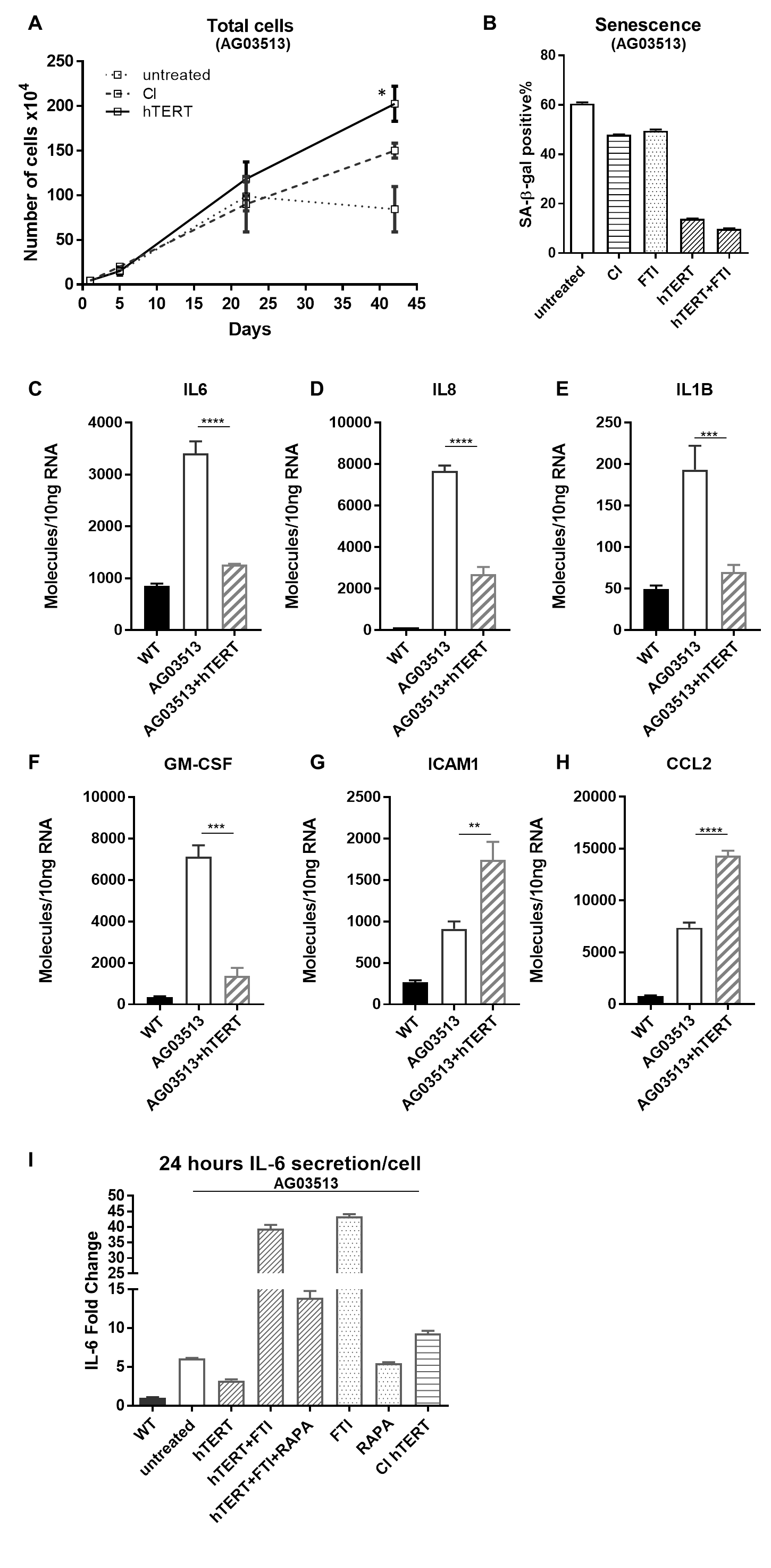


**Supplemental F7. Effect of transient telomerase expression on cells derived from a second HGPS patient.** AG03513 cells were treated three times at 48 h intervals followed by lonafarnib (FTI; 1μM) and everolimus (RAPA; 10nM) treatment. (A)Total cell numbers were monitored for 45 days. *n=2*; **P*<0.05; (B) SA-β-gal staining. (C-H) The mRNAs of inflammatory cytokines were examined by droplet PCR 14 days after the 3 treatments with HPLC grade hTERT mRNA. (D) IL-6 ELISA was performed on the 17^th^ day after the 3^rd^ treatment and normalized to an age matched WT control.


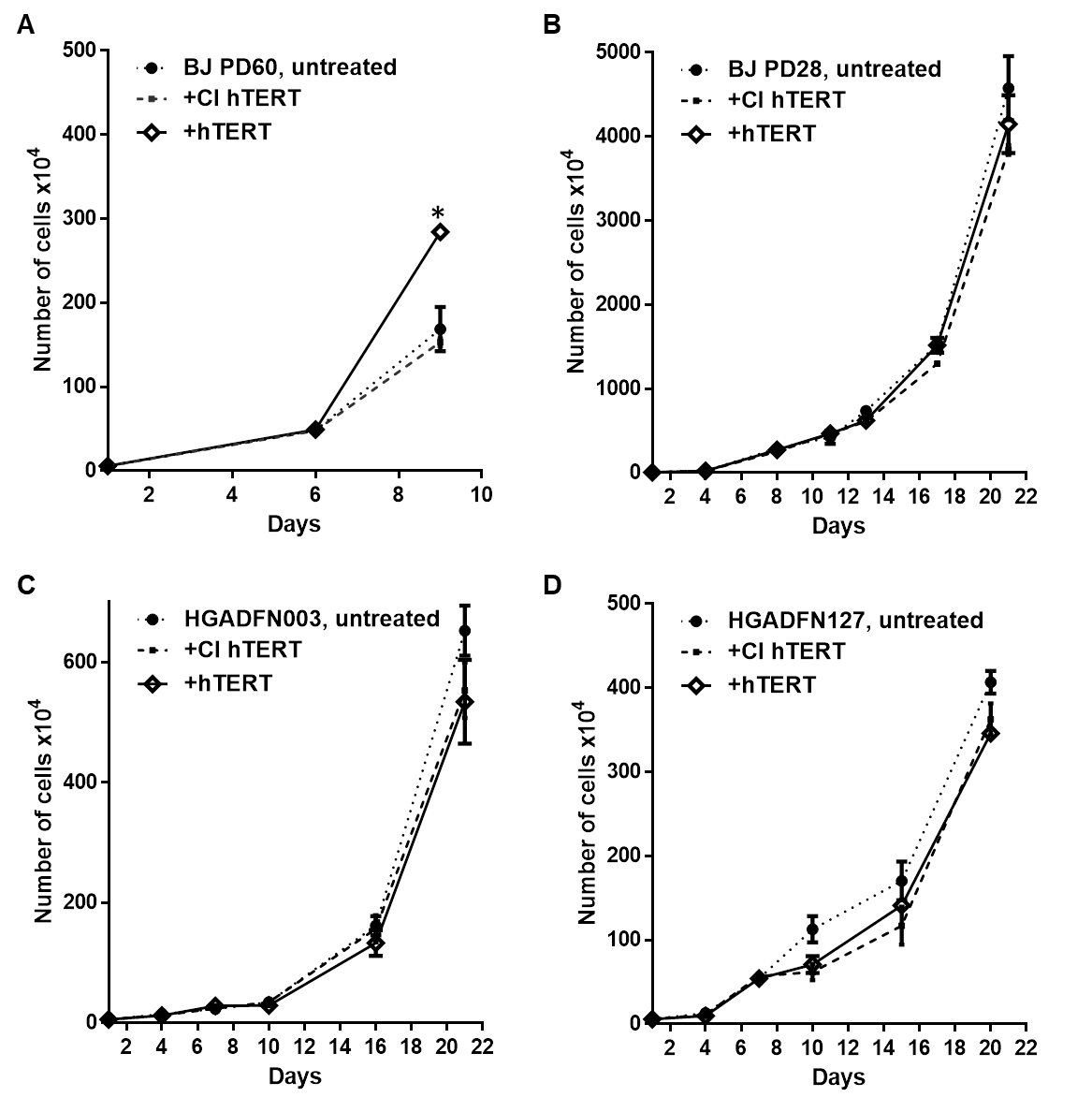


**Supplemental F8. Short exposure to hTERT mRNA maintains long-term proliferation of cells with short telomeres but not long telomeres**. Short telomere BJ(PD60), long telomere BJ (PD28), HGADFN127 and HGADFN003 cells were treated with hTERT mRNA (1 µg/ml) every 48 hours for 3 times**.** **P < 0.05.*


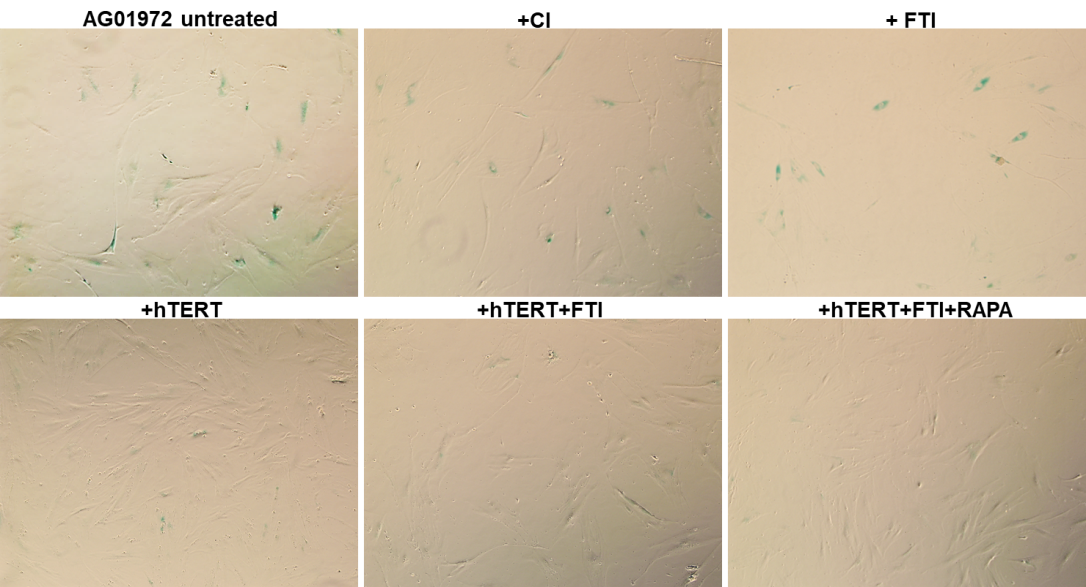


**Supplemental F9. SA- β-gal staining of AG01972 cells.** hTERT mRNA reduces the percent of SA- β-gal positive cells


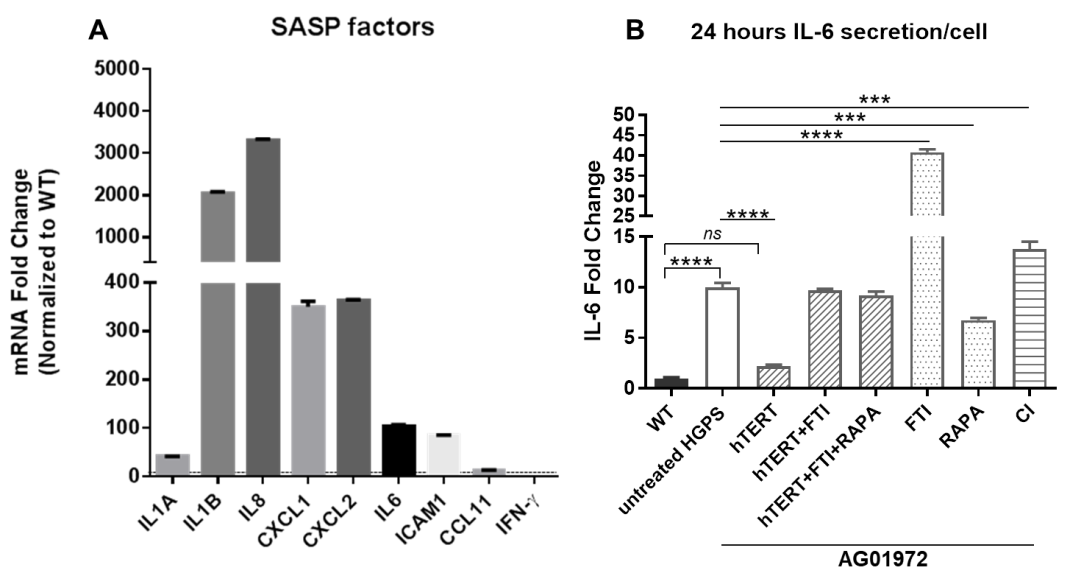


**Supplemental F10. Transient expression of telomerase reverses HGPS SASP.** (A) AG11498 progeria cells and WT cells were assayed for cytokine expression using Human NF-ĸB signaling array (Qiagen). Gene expression was normalized to WT. Error bar indicates SD; *n=2*. (B) After 3 transfections with hTERT mRNA (HPLC grade), AG01972 cells were treated with lonafarnib (FTI; 1μM) or lonafarnib and everolimus (RAPA; 10nM) for two weeks. IL-6 ELISA was performed 17 days after the 3^rd^ treatment. Error bar indicates SD; n=3; ****P* <0.001; *****P* <0.0001.

**
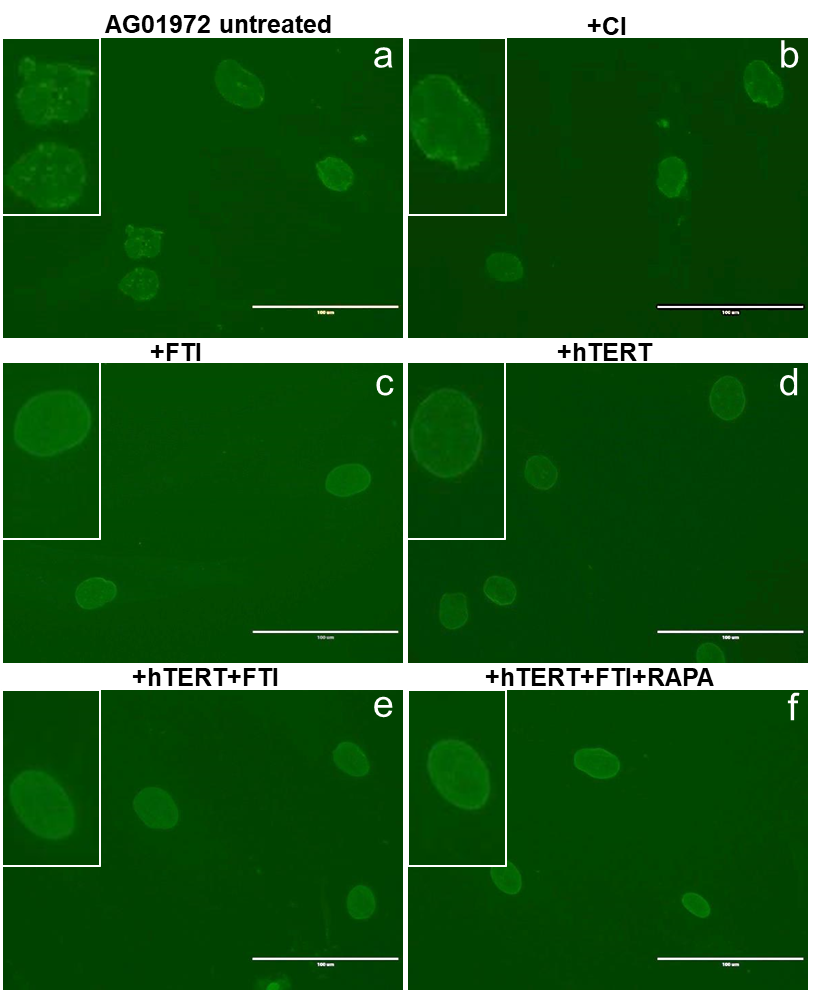
**

**Supplemental F11. Nuclear morphology for Figure 3E.** hTERT mRNA provides a more normal nuclear morphology.


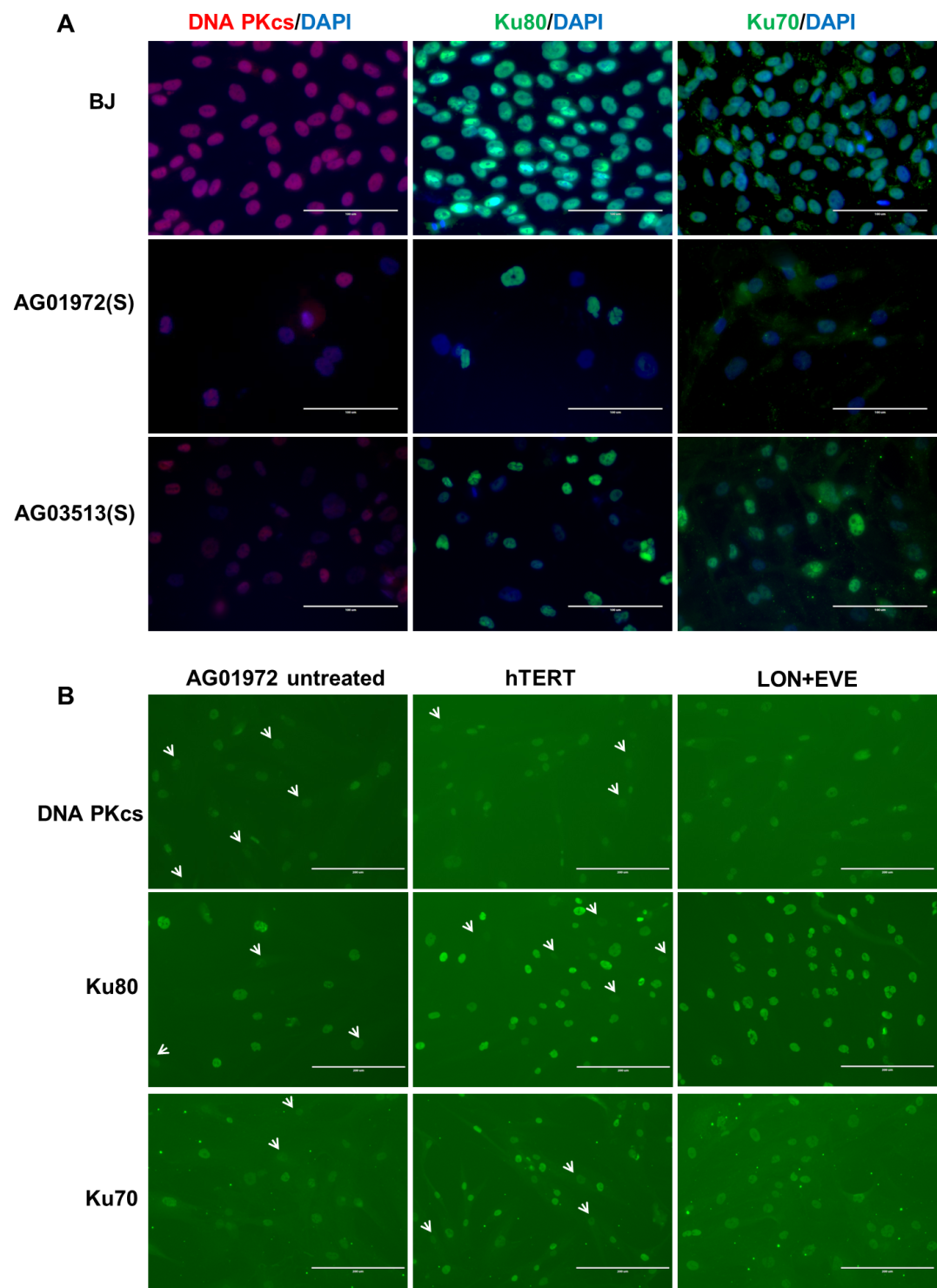


**Supplemental F12. Transient telomerase expression partially rescues DNA PKcs, Ku70 or Ku80 in HGPS.** (A) WT and HGPS cells were stained by anti-DNA PKcs (purple), anti-Ku80 (green) and anti-Ku70 (green) and DAPI (blue). (B) AG01972 (P21) cells were treated with hTERT or CI hTERT mRNA (1 µg/ml) every 48 hours for 3 times**.** Cells were cultured one week, followed by one-week of treatment with lonafarnib (FTI, 1μM) and/or everolimus (EVE; 10nM). White arrows indicated low staining cells.


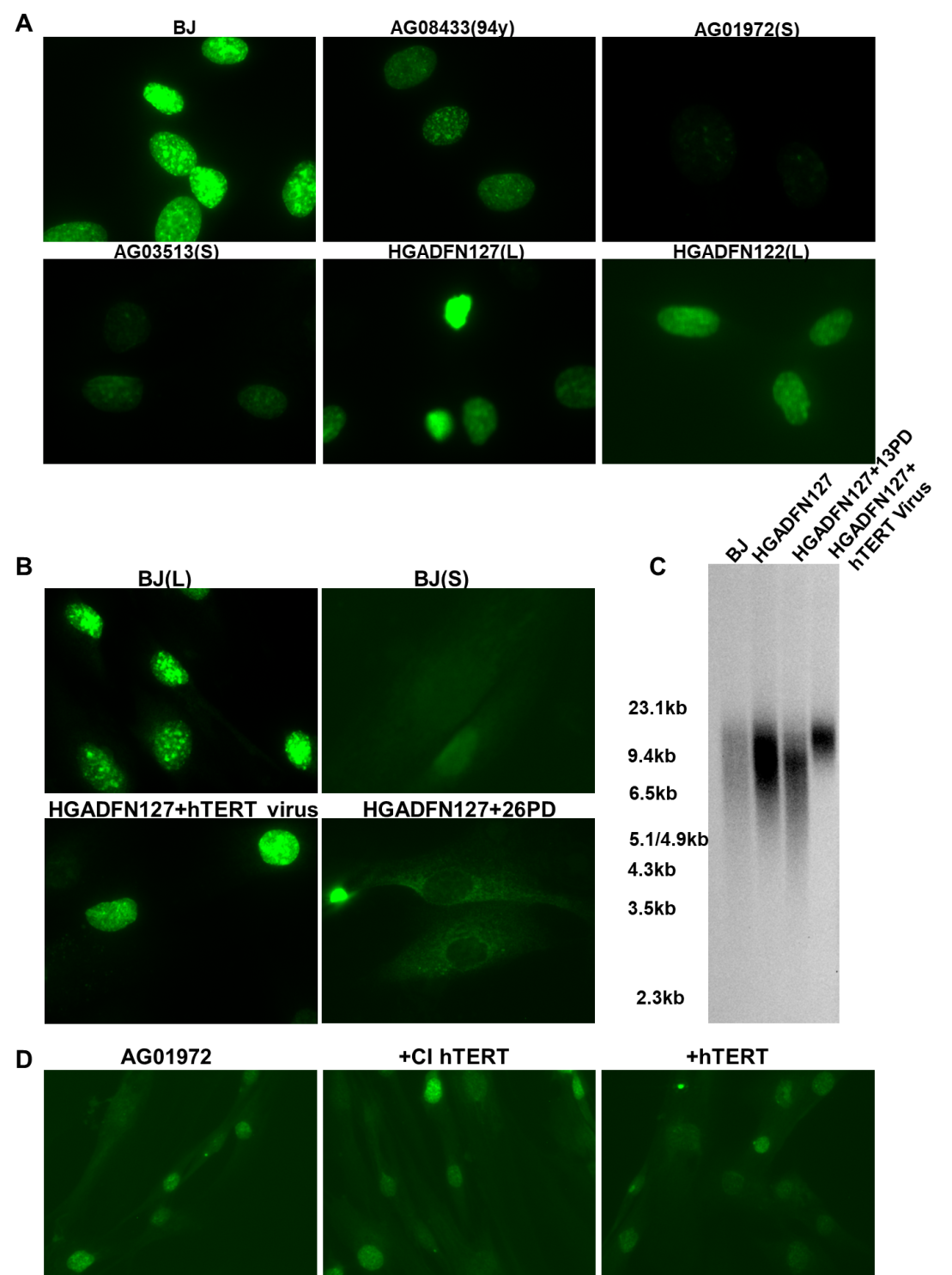


**Supplemental F13. Loss of heterochromatin in progeria cells is associated with telomere shortening.** (A) BJ(PD28) and progeria cells were stained by anti-H3K9me3. (B) BJ or HGADFN127 cells from isogenic clones with short and long telomeres were stained by anti-H3K9me3. (C) TRF analysis of telomere length in HGADFN127 stably expressing telomerase or by continuous passage. (D) AG01972 were treated with hTERT or CI hTERT mRNA (1 µg/ml) every 48 hours for 3 times. Cells were stained 14 days after the last treatment by anti-H3K9me3.

**Supplemental Table1. Profile of HGPS patients derived fibroblast cell lines**

| **Cell Line ID** | **Age (At Sampling)** | **Gender** | **Mutation** | **Passage#** | **Resource** |
| --- | --- | --- | --- | --- | --- |
| AG01972 | 14 year | F | LMNA Exon 11, c.1824C>T | 15 (28.5PDs) | Coriell Institute |
| AG03513 | 13 year | M | LMNA Exon 11, c.1824C>T | 9(15PDs) | Coriell Institute |
| AG11498 | 14 year | M | LMNA Exon 11, c.1824C>T | 5(9.51PDs) | Coriell Institute |
| AG11513 | 8 year | F | LMNA Exon 11, c.1824C>T | 6(9.4PDs) | Coriell Institute |
| AG03199 | 10 Year | F | LMNA Exon 11, c.1824C>T | 6(6PDs) | Coriell Institute |
| AG06917 | 3 Year | M | LMNA Exon 11, c.1824C>T | 3(39.95PDs) | Coriell Institute |
| AG16102 | 69 year | M | WT | 6(8PDs) | Coriell Institute |
| AG08470 | 10 year | F | WT | 4(5PDs) | Coriell Institute |
| BJ | newborn | M | WT | 3(4PDs) | ATCC |
| HGADFN003 | 2 yrs | M | LMNA Exon 11, c.1824C>T | 7 | PRF |
| HGADFN122 | 5 yrs | F | LMNA Exon 11, c.1824C>T | 7 | PRF |
| HGADFN127 | 3 yrs 9 mos | F | LMNA Exon 11, c.1824C>T | 7 | PRF |
| HGADFN143 | 8 yrs 10 mos | M | LMNA Exon 11, c.1824C>T | 7 | PRF |
| HGADFN155 | 1 yr 2 mos | F | LMNA Exon 11, c.1824C>T | 7 | PRF |
| HGADFN164 | 4 yrs 8 mos | F | LMNA Exon 11, c.1824C>T | 7 | PRF |
| HGADFN167 | 8 yrs 5 mos | M | LMNA Exon 11, c.1824C>T | 6 | PRF |
| HGADFN169 | 8 yrs 6 mos | M | LMNA Exon 11, c.1824C>T | 5 | PRF |
| HGADFN178 | 6 yrs 11 mos | F | LMNA Exon 11, c.1824C>T | 7 | PRF |
| HGADFN271 | 1 yr 3 mos | M | LMNA Exon 11, c.1824C>T | 6 | PRF |
| HGADFN367 | 3 yrs 0 mos | F | LMNA Exon 11, c.1824C>T | 7 | PRF |
| HGMDFN090 | 37 yrs 10 mos | F | WT | 10 | PRF |
| HGMDFN368 | 31 yrs 7 mos | F | WT | 7 | PRF |
